# Sensory Acceptance, Appetite Control and Gastrointestinal Tolerance of Yogurts Containing Coffee-Cascara Extract and Inulin

**DOI:** 10.3390/nu12030627

**Published:** 2020-02-27

**Authors:** Maite Iriondo-DeHond, Amaia Iriondo-DeHond, Teresa Herrera, Adriana Maite Fernández-Fernández, Carlos Oscar S. Sorzano, Eugenio Miguel, María Dolores del Castillo

**Affiliations:** 1 Instituto Madrileño de Investigación y Desarrollo Rural, Agrario y Alimentario (IMIDRA), N-II km 38,200, 28800 Alcalá de Henares, Spain; maite.iriondo@madrid.org (M.I.-D.); eugenio.miguel@madrid.org (E.M.); 2 Instituto de Investigación en Ciencias de la Alimentación (CIAL) (UAM-CSIC), C/ Nicolás Cabrera, 9, Campus de la Universidad Autónoma de Madrid, 28049 Madrid, Spain; amaia.iriondo@csic.es (A.I.-D.); teresa.herrera@csic.es (T.H.); 3 Departamento de Ciencia y Tecnología de Alimentos, Facultad de Química, Universidad de la República, General Flores 2124, Montevideo 11800, Uruguay; afernandez@fq.edu.uy; 4 Centro Nacional de Biotecnología (CNB-CSIC), C/ Darwin, 3, 28049 Madrid, Spain; coss@cnb.csic.es

**Keywords:** carbohydrate metabolism, coffee byproduct, coffee-cascara, gastrointestinal-tolerance, inulin, nutritional trial, satiety, organoleptic properties

## Abstract

The improvement of the nutritional quality of dairy foods has become a key strategy for reducing the risk of developing diet-related non-communicable diseases. In this context, we aimed to optimize the concentration of inulin in combination with 10 mg/mL of coffee-cascara extract in yogurt while considering their effect on appetite control, gastrointestinal wellbeing, and their effect on the sensory and technological properties of the product. For this purpose, we tested four coffee-cascara yogurt treatments in a blind cross-over nutritional trial with 45 healthy adults: a coffee-cascara yogurt without inulin (Y0) and coffee-cascara yogurts containing 3% (Y3), 7% (Y7), and 13% (Y13) of inulin. The ratings on sensory acceptance, satiety, gastrointestinal tolerance, and stool frequency were measured. Surveys were carried out digitally in each participant’s cellphone. Yogurt pH, titratable acidity, syneresis, and instrumental texture were analyzed. Inulin addition increased the yogurt’s firmness and consistency. Y13 achieved significantly higher overall acceptance, texture, and taste scores than Y0 (*p* < 0.05). Y3 presented similar gastrointestinal tolerance to Y0. However, 7% and 13% of inulin produced significant (*p* < 0.05) bloating and flatulence when compared to Y0. The appetite ratings were not significantly affected by the acute intake of the different yogurts. Overall, Y3 was identified as the formulation that maximized nutritional wellbeing, reaching a “source of fiber” nutritional claim, without compromising its technological and sensory properties.

## 1. Introduction

Suboptimal diets are one of the leading risk factors for the prevalence of non-communicable diseases, which are responsible for 71% of all deaths globally [1]. Government measures are emerging for improving the nutritional quality of the food supply because the epidemic of diet-related non-communicable diseases is forecast to increase in coming years. These measures are directed at actors in the food supply chain (producers, processors and retailers) to reduce the levels of critical nutrients, such as salt, sugar, or fat, and promote functional and healthier foods [2,3]. Dairy products are one of the food groups for which reformulation policies are prioritized due to the excessive amount of added sugars [2].

Functional foods are those that have a potentially positive effect on health beyond basic nutrition, promoting optimal health conditions and reducing the risk of non-communicable diseases [4]. Several authors have stated the need to develop functional foods with a multidisciplinary approach that combines principles from sensory science, nutrition physiology, ingredient technology, and texture design [4,5]. Following this multidisciplinary approach, we proposed the development of functional yogurts without added sugars that include ingredients (coffee-cascara and inulin) that are involved in appetite control.

The industrial interest on the use of coffee-cascara, which is a byproduct of the coffee industry, as a food ingredient, has exponentially increased over the last years [6]. Coffee-cascara is an officially authorized food in the US and it has become a popular ingredient in the American beverage industry. In Europe, coffee-cascara is considered to be a novel food and must comply with Regulation (EU) 2015/2283 to achieve authorization by the European Food Safety Authority (EFSA) before it can be used in foods in the European market. Evidence on the functional potential (antioxidant and antidiabetic properties) and safety (pesticide, mycotoxin, acrylamide, and acute toxicity experiments) of coffee-cascara as a sustainable food ingredient is already being gathered for its authorization [7]. This by-product has shown α-glucosidase inhibition properties (Figure S1), which may affect the regulation of carbohydrate metabolism and enhance gut satiety signals [8,9]. On the other hand, inulin is a non-digestible carbohydrate, a dietary fiber, with a potential role in appetite control. This effect has been linked to inulin fermentation by colonic bacteria, which produces short-chain fatty acids that are also involved in the regulation of gut hormones that are implicated in satiety and appetite control (GLP-1, PYY, and ghrelin) [10,11,12]. The combination of both ingredients on the modulation of appetite control has not been previously studied.

Inulin has been widely used in dairy products as a texturizing agent for improving the product’s mouthfeel, as it interaction with the dairy matrix creates a creamy texture that is comparable to that of fat [13,14]. Other motivations for using inulin in the dairy industry are focused on helping consumers to reach the dietary fiber daily intake recommended by regulatory bodies (25 g dietary fiber per day) [15]. In this sense, inulin concentrations of 3% or 6% are necessary for achieving a “source of fiber” or “high in fiber” nutritional claim, respectively [16]. However, inulin consumption might have some dose-related undesirable effects due to their natural osmotic potential and/or excessive fermentation [17]. Therefore, inulin concentrations should be based on a compromise between their nutritional and technological properties, without producing any negative secondary gastrointestinal effects.

Based on these premises, we aimed to optimize the concentration of inulin in combination with 10 mg/mL of coffee-cascara extract in yogurt while considering their effect on appetite control, gastrointestinal wellbeing, and their effect on the sensory and technological properties of the product. For this purpose, we tested four coffee-cascara yogurt treatments in a blind cross-over nutritional trial with 45 healthy adults: a coffee-cascara yogurt without inulin (Y0) and coffee-cascara yogurts containing 3% (Y3), 7% (Y7), and 13% (Y13) of inulin. This multidisciplinary approach stands as a risk-balance assessment that contemplates the nutritional, sensory, and technological dimensions of the new food product development process.

## 2. Materials and Methods

### 2.1. Test Foods

The test yogurts were made at the Instituto de Investigación en Ciencias de la Alimentación (CIAL, UAM-CSIC) while using UHT whole cow milk (Unicla, Feiraco, Spain), inulin (Orafti®GR, Beneo, Belgium) and coffee-cascara from Arabica species and Colombian origin (Supracafe, Spain). Cascara extract was produced as described in the patent WO 2013/004873 [18]. 

Four test coffee-cascara yogurts were produced: a control cascara yogurt without inulin (Y0), cascara yogurt with 3% inulin (Y3), cascara yogurt with 7% inulin (Y7), and cascara yogurt with 13% inulin (Y13). Coffee-cascara extract was used due to its inhibitory properties against the enzyme α-glucosidase (IC50 = 0.70 ± 0.10 mg/mL) (Figure S1). The α-glucosidase inhibitory capacity of coffee-cascara extract was analyzed following the methodology that was described by Berthelot & Delmotte [19] and Geddes & Taylor [20], with modifications [21].

For the test yogurt elaboration, milk (3.6% fat, 3.1% protein, and 4.8% sugar) was put in a vat and heated up to 45 °C to inoculate the starter culture YO-MIX 300 (Danisco), containing *Streptococcus thermophilus* and *Lactobacillus delbrueckii subsp. bulgaricus*. Inulin was added at 3 g/100 mL, 7 g/100 mL, or 13 g/100 mL in Y3, Y7 and Y13, respectively. All of the yogurts contained cascara extract at a final concentration of 10 mg/mL. Extract concentration was selected while using a focus group of 10 volunteers who tested yogurts containing cascara extract within a range of 3–15 mg cascara/mL yogurt. After the addition of the ingredients, milk was stirred and separated into pots of 125 g. Individual pots were incubated at 45 °C during 5 h until the pH reached approximately 4.5. The samples were stored at 4 °C. Yogurt samples for pH, titratable acidity, texture, and syneresis analyses were separately elaborated in three independent sets and analyzed on the following day. Yogurt samples for the nutritional trial were elaborated in advance to each session independently. Therefore, all of the participants tasted yogurts with two or three days of shelf life.

### 2.2. Physicochemical Characterization of Test Yogurts: pH, Titratable Acidity, Texture, and Syneresis Analyses

The measurements of pH were taken with a Hanna Instruments HI5521 pH meter. Titratable acidity was determined according to ISO:RM 2012 and expressed as g lactic acid/ 100 g of yogurt. Yogurt instrumental texture was analyzed while using a TA.XTplus Texture Analyzer (Stable Micro Systems, Godalming, UK). A back-extrusion test was carried out while using a cylindrical stainless-steel probe (35 mm diameter). Yogurts for texture analysis were made directly into cylindrical containers (50 mm diameter and 50 mm high), so that their solid structure would be kept intact prior to the texture analysis. The probe penetrated the sample to a depth of 10 mm at 1 mm/s. The firmness (N) and consistency (Ns) were calculated from the deformation curves while using the Exponent software (Stable Micro Systems, Godalming, UK). Measurements were performed in triplicate.

Yogurt syneresis was calculated by centrifugation [22]. The results were expressed in percentage, according to the following equation:

Syneresis (%) = [expelled whey (g)/ yogurt mass (g)] ×100

### 2.3. Nutritional Study

#### 2.3.1. Participants and Ethical Aspects 

Healthy participants were recruited through advertising at the Universidad Autónoma de Madrid and surrounding areas. The inclusion criteria included apparently healthy participants, male or female, age ≥ 18 and ≤ 60 years old. The exclusion criteria were: pregnancy or breastfeeding, having food allergies, lactose intolerance, milk protein allergy, and diagnosed gastrointestinal disorders/diseases.

This study was granted a favorable ethical opinion from the Spanish National Council Ethics Committee (reference: 034/2017) and it was registered under http://clinicaltrials.gov as NCT03539146. The study was conducted in accordance with the ethical standards that were laid down by the 1964 Declaration of Helsinki and in accordance with Good Clinical Practice guidelines. All of the participants gave written informed consent to participate in the study and they were aware of the possibility of withdrawing from the study at any time they desired. 

The recruiting process resulted in the involvement of forty-six participants in the study, 26 females and 20 males. One participant withdrew from the study due to severe gastrointestinal symptoms. The participants ranged in age from 20 to 57 years, with a median of 28.5 years. The participants had a median Body Mass Index (BMI) of 22.8, with four (9%) underweight (BMI < 18.5), 28 (62%) normal weight (BMI ≥ 18.5 and < 25), 11 (24%) over- weight (BMI ≥ 25 and < 30), and two (4%) obese (BMI ≥ 30).

#### 2.3.2. Study Design and Protocol

This study used a blind, crossover design, including four treatments of yogurts containing coffee-cascara extract and different doses of inulin (0, 3, 7, and 13%). Four sequences of intervention groups were used following a completely balanced Latin square design to avoid first-order carryover effects. 

Over a period of two weeks, each volunteer visited the test facility on four occasions, with a minimum of 48-hour washout period between visits. The participants were asked not to change their dietary patterns or physical activity routine for the duration of the trial. On each test day, the participants were asked to arrive at the Centro de Biología Molecular cafeteria at 08.45 in fasting conditions to have a standardized breakfast. The standardized breakfast (300 Kcal) consisted on a 40 g toasted white bread baguette with 8 mL of olive oil and salt, 150 mL fresh orange juice, and 200 mL of tea or coffee. At 11.15, a test yogurt was given to each participant at the Instituto de Investigación en Ciencias de la Alimentación (CIAL, UAM-CSIC), together with a glass of water (150 mL). The participants were given the choice to work on a laptop at the Institute’s facilities or at their own facility, as all of the volunteers worked on campus. However, they were instructed not to eat or drink between meals, with the exemption of small amounts of water. 

The participants completed a baseline appetite rating at 09.00 prior to the consumption of breakfast and they were given 15 minutes to eat it. Questions on satiety were then asked every 30 minutes until the consumption of the yogurt at 11.15. The participants had 15 minutes to eat the test yogurt (125 g) and respond to a sensory acceptance questionnaire of the product. After consumption of the test yogurt, the participants continued to answer questions on satiety every 30 minutes until lunch time (13.30). At that time, the participants were also asked to answer a gastrointestinal symptom questionnaire (two-hours after test yogurt ingestion). The gastrointestinal symptoms were also evaluated with an online survey 24 h after yogurt ingestion. 

#### 2.3.3. Sensory Analysis

Participant’s acceptance of test yogurts was assessed while using a nine-point hedonic scale that ranged from “1-dislike extremely” to “9-like extremely”. The attributes evaluated included “overall liking”, “visual appearance”, “smell”, “texture”, and “taste”. The data were collected on a five-point just-about-right (JAR) scale to measure the appropriateness of the level of the following attributes: “creaminess”, “sweetness”, “vegetal or fruity flavor”, and “lactic flavor”. The JAR scale ranged from 1 (“not enough at all”) to 5 (“Far too much”). Liking and JAR data were used for a penalty analysis to study the relation between the rankings on the JAR scale and the results in the liking scores for the different attributes.

#### 2.3.4. Appetite Ratings 

Subjective appetite ratings were electronically measured on each of the participants’ cell phone with the use of 100 mm VAS (RedJade Sensory Software, Martinez, CA, USA). The participants were asked to set alarms on their cell phones at the indicated times (every 30 minutes) and the answers were checked online in real time. The appetite rating profile included measures of hunger, satiety, fullness, thirst, and desire to eat something fatty, salty, sweet, or savory. The scale was anchored at 0 mm (“nothing at all”) and at 100 mm (“a large amount”).

#### 2.3.5. Gastrointestinal Wellbeing Measurements

Gastrointestinal symptoms were assessed two and 24-hours after test yogurt intake. The participants were asked to compare their symptoms to how they normally felt in a scale from 0 to 3 [23]: “0-no or habitual occurrence of symptom”, “1-slightly more than usual”, “2-much more than usual”, and “3-exceptionally more than usual”. The gastrointestinal profile included ratings on abdominal bloating, nausea, abdominal pain, flatulence, diarrhea, constipation, and stomach rumbling. Individual gastrointestinal scores for each symptom were collected and the total score of gastrointestinal symptoms was calculated as the sum of the individual scores for each treatment. The participants were also asked to record the stool frequency and consistency in accordance with the Rome III clinical designation (constipation, normal, or diarrhea) after 24 hours.

### 2.4. Statistical Analysis 

The minimum sample size (*n* = 34) was estimated through a power analysis for the detection of 0.5 variation in gastrointestinal symptoms, with power of 80% and alpha = 0.05. A one-factor ANOVA with Tukey’s HSD post-hoc test was used to analyze textural differences among yogurt samples. The randomization sequences were analyzed while using one-factor analysis of variance to examine whether there was evidence of carry-over effects among treatments. A Chi-square test was used to compare the frequency of gastrointestinal symptoms, stool frequency, and stool consistency types that are associated to the ingestion of the different cascara yogurt and inulin treatments (Y3, Y7, Y13) to those of the cascara yogurt without inulin (Y0). These analyses were performed while using R software version 3.5.1. A two-way, repeated measures ANOVA with Tukey’s HSD post-hoc test was used to analyze satiety scores, with time as the within-subject factor. Satiety statistical analyses were performed while using IBM SPSS Statistics version 24. Hedonic data were analyzed with a non-parametric test, as the data were not normally distributed. A Friedman test followed by multiple pairwise comparisons while using Nemenyi’s procedure was used for determining differences between treatments. The results from the JAR and liking surveys were used to determine the drop in overall liking that was associated with a deviation from the JAR for each attribute. Sensory analyses were conducted in XLStat-Sensory version 2018.6.

## 3. Results

### 3.1. Physicochemical Characterization of Yogurts

**Figure 1:**
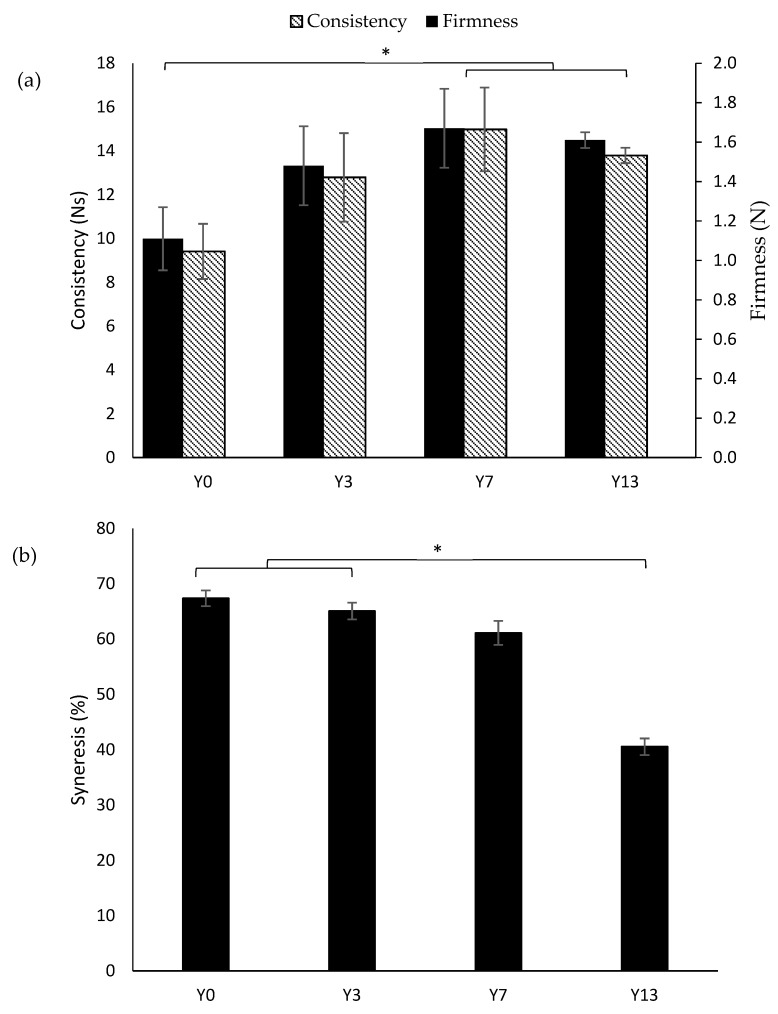
(**a**) Instrumental firmness (N) and consistency (Ns) and (**b**) syneresis (%) of yogurt with cascara extract (Y0), yogurt with cascara extract and 3% inulin (Y3), yogurt with cascara extract and 7% inulin (Y7), and yogurt with cascara extract and 13% inulin (Y13). The asterisk indicates significant differences (Tukey test, * *p* < 0.05).

Table 1 and Figure 1 show the effect of coffee-cascara and inulin addition on the pH, titratable acidity, texture, and syneresis properties of yogurt. The measures of pH did not differ between the formulated yogurts, whereas titratable acidity was significantly lower (*p* < 0.05) in Y13 as compared to Y3. The addition of 7 % and 13 % of inulin in coffee-cascara yogurts significantly increased (*p* < 0.05) the instrumental firmness and consistency of Y7 and Y13 when compared to Y0 (Figure 1a). In relation to the effect of inulin on the yogurts’ syneresis, inulin addition significantly reduced (*p* < 0.05) syneresis levels in Y13 when compared to Y0 and Y3 (Figure 1b).

### 3.2. Sensory Quality and Gastrointestinal Effects of Yogurt Intake 

#### 3.2.1. Sensory Quality

**Figure 2:**
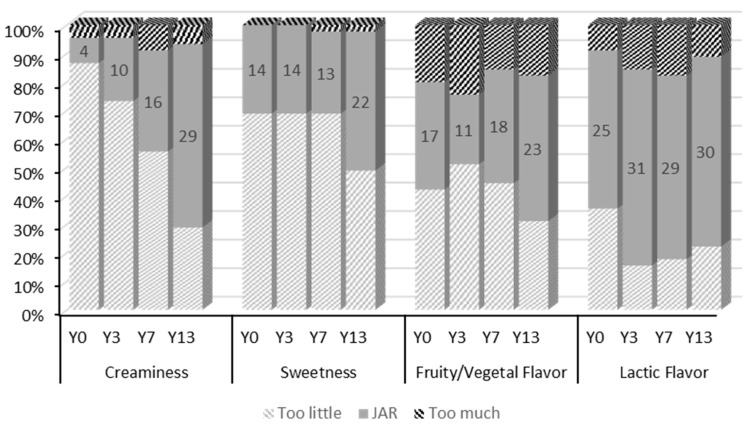
Frequency distribution in percentage of consumer responses for Just-About-Right scores for each dimension (Too little, JAR, Too much) and each attribute. The number of consumers out of the total (*n* = 45) that rated an attribute as “Just About Right” is indicated in each column.

Table 2 shows the results from the hedonic test. Overall liking was significantly higher (*p < 0.001*) in Y13 than in Y0. Texture and taste were other parameters influenced by the addition of inulin. Texture was significantly better accepted in Y13 (*p < 0.001*) when compared to the rest of the yogurt formulations. Y13 also achieved significantly higher scores in taste (*p* < 0.05) than Y0 and Y3. JAR scale results showed that less than 65% of the consumers stated that “creaminess”, “sweetness”, and “fruity/vegetable flavor” were in the ideal-JAR point for all yogurt treatments (Figure 2). However, increasing doses of inulin raised the number of respondents in the JAR point for “creaminess” and “sweetness”. Over 65% of the participants rated “lactic flavor” of Y3, Y7, and Y13 as JAR. The penalty analysis showed the mean drop in liking scores for the attributes that had a significant negative effect (*p* < 0.05) and an occurrence higher than 20% of cases. These parameters identified that too little “lactic flavor” produced a significant (*p* < 0.01) mean drop in the overall liking of 1.05 in Y0 and of 1.13 in Y13.

#### 3.2.2. Appetite Ratings

**Figure 3:**
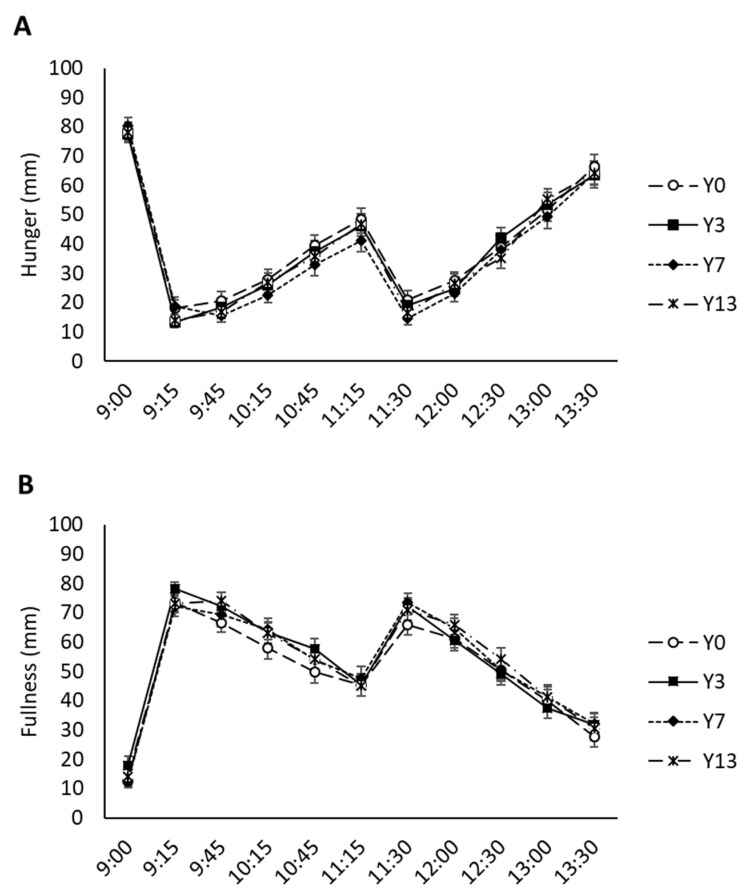
VAS scores of hunger (**A**) and fullness (**B**) rated from the morning meeting in fasting conditions before the standard breakfast (9:00 h) to prior to lunch time (13:30 h).

Figure 3 shows VAS ratings for hunger and fullness. Hunger and fullness values of the same order of magnitude were found after breakfast and yogurts intake. No significant differences were found among the cascara yogurt treatments with increasing doses of inulin concerning the ratings of hunger, satiety, fullness, thirst, and desire to eat something fatty, salty, sweet, or savory at different time points. However, a non-statistically significant trend on the combined effect of cascara and inulin in appetite control was observed. This trend suggests higher hunger and lower fullness ratings after the intake of Y0 when compared to other yogurts containing both ingredients (coffee-cascara extract and inulin).

#### 3.2.3. Gastrointestinal Tolerance

The randomization sequences were not significantly different from one another (*p* > 0.05) while using one-factor analysis of variance, indicating that there was no evidence of carry-over effects among treatments. The occurrence of gastrointestinal symptoms was only reported between 2h and 24h after treatment intake (Table 3). Bloating was the most experienced symptom, with 49% and 58% of participants reporting occurrence in Y7 and Y13. Flatulence was the second most experienced symptom. Most of the participants who experienced these only reported mild occurrences, as the individual symptom scores remained in a low range (0.58–0.82 out of 3). The consumption of Y7 significantly increased the occurrence of bloating (*p* < 0.05) and flatulence (*p* < 0.01) as compared to Y0. In relation to Y13, a significant increase in the occurrence of bloating (*p* < 0.01), flatulence (*p* < 0.01), abdominal pain (*p* < 0.05), and gastrointestinal rumbling (*p* < 0.05) was observed when compared to Y0. The consumption of Y3 did not significantly increase (*p* > 0.05) the occurrence of individual gastrointestinal symptoms as compared to Y0. The total gastrointestinal symptom scores of yogurt treatments increased 0.18, 1.07, and 1.62 in Y3, Y7, and Y13, when compared to the basal Y0 total score.

Stool frequency did not significantly increase (*p* > 0.05) within the 24 h following the intake of yogurts containing cascara extract and inulin (Table 4). The frequency of stools with a consistency that is similar to diarrhea slightly increased after the consumption of yogurts containing higher concentrations of inulin (Y7 and Y13), although the difference was not significant when compared to the frequency of diarrheic stools reported in the cascara yogurt without inulin (Y0). 

## 4. Discussion

The firmness and consistency of coffee-cascara yogurts were improved by increasing the concentrations of inulin (Figure 1). Moreover, the addition of inulin decreased the yogurts syneresis, which refers to the serum release from the gel matrix and it is regarded as a technological defect in set yogurts. Similar results on syneresis reduction and increased firmness were observed in set yogurts with increasing doses of inulin (0 to 4%) [24]. Yogurt syneresis is affected by pH and titratable acidity levels [25]. This relation is observed by the significantly lower levels of syneresis (Figure 1b) and titratable acidity of Y13 (Table 1) as compared to Y3 (*p* < 0.05). 

The sensory analysis conducted with the nutritional trial participants (*n* = 45) provided preliminary information on the acceptance of yogurts containing coffee-cascara and inulin, and on the properties of inulin as a texture and flavor enhancer. As expected, the results on physicochemical characterization are in line with those on sensory analysis. The hedonic scores for “taste” and JAR data on “fruity or vegetable flavor” in yogurts suggest that the unique flavor of coffee-cascara, although unfamiliar, was well accepted. The addition of increasing levels of inulin in the yogurts showed a positive tendency, increasing the overall liking and acceptance scores for all attributes. JAR scales measured the appropriateness of the level of specific attributes and they were used as guidance for product reformulation. The number of participants that rated “creaminess” as “just-about-right” increased with increasing doses of inulin, which confirmed the texture modifying properties of inulin in yogurt. Industrial food applications of long chain inulin mainly include texturizing, bulking, and fat replacing functions [13]. 

The results on the appetite measures after consumption of the coffee-cascara yogurt treatments showed that increasing concentrations of inulin in yogurt did not modify ratings on hunger, fullness, satiety, thirst, and desire to eat something fatty, salty, sweet, or savory. Similarly, previous studies showed no differences in the hunger ratings of healthy young adults between yogurts with or without 6 g of inulin in a one-time consumption basis [26]. Other studies in which inulin intake showed an effect on appetite control include two-weeks of repeated consumption of symbiotic yogurts containing 4 g of inulin, which reduced the reported energy intakes [27]; and, eight-day consumption of yogurts containing 6 g of inulin, which significantly lowered the “desire to eat” and “prospective food consumption” ratings [28]. There is no previous research on the effect of coffee-cascara on appetite modulation. Future studies are needed to determine whether chronic consumption of the developed product might influence appetite control.

A high inulin level may compromise the gastrointestinal tolerance of the food [29]. The effect of coffee-cascara components on that is unknown. The assessment of the risk-benefit balance of using inulin and coffee-cascara to develop a novel food product that is well tolerated is critical to secure product intake and adherence by consumers. Therefore, yogurts containing coffee-cascara and inulin were evaluated alone in acute conditions to simulate the action of eating a yogurt as a mid-day snack. The selection of the optimal inulin concentration for the development of functional coffee-cascara yogurts was based on the condition that no significant differences should be observed in the gastrointestinal tolerance between the yogurt without inulin (Y0) and the yogurts containing inulin (Y3, Y7, Y13). Our results show that this threshold was only true for the yogurt containing 3% of inulin (Y3), which corresponded to 3.75 g of inulin per portion (125 g of yogurt), which would allow for a “source of fiber” nutritional claim. Data are in line with previous findings on the recommended inulin consumption doses, which described that intakes below 10 g may cause mild gastrointestinal symptoms, intakes between 10–15 g of inulin per day may be tolerated by most individuals with only mild effects, and that intakes of more than 20 g per day may increase the occurrence and severity of the symptoms [30].

Although this approach might appear conservative, it is better to identify the upper intake levels to minimize the risk of undesirable gastrointestinal effects while maximizing the potential health benefits of inulin consumption. This is because gastrointestinal symptoms may affect the perception of the well-being by consumers and their acceptance of food products containing indigestible carbohydrates, which could put their marketability at risk. The addition of 7% inulin resulted in a significant occurrence of bloating and flatulence when compared to Y0. At higher doses (13%), more painful symptoms occurred, such as abdominal cramps, also accompanied by bloating, flatulence, and gastrointestinal rumbling. These results are in accordance with previous reports on the order of occurrence of individual gastrointestinal symptoms associated with increasing intake of indigestible carbohydrates [17]. Diarrhea is usually the last intolerance symptom occurring with high doses of indigestible carbohydrates. In our study, none of the yogurts containing inulin produced significant differences in stool frequency or in the occurrence of different stool consistencies when compared to Y0.

The occurrence of gastrointestinal symptoms may have been generally intensified in our study by the fact that inulin was added in a semisolid food matrix (yogurt) without the simultaneous intake of other foods, which can help to increase tolerance [31]. Yogurt might travel through the gastrointestinal tract and be absorbed relatively more quickly than more solid foods, which are generally better tolerated than liquid structures [32]. Previous studies have reported that inulin intake in acute conditions was well tolerated at 5, 9, and 10 g when administered together with breakfast [29,33,34]. Additionally, studies that spread out the dose of fiber throughout the day also observed improved inulin tolerance [35]. Therefore, it might be possible that coffee-cascara yogurts containing amounts of inulin above 3% could be better tolerated if the yogurt is incorporated as part of a meal. 

## 5. Conclusions

To our knowledge, this is the first time that the coffee-cascara extract and inulin have been combined to assess their effects on appetite control and gastrointestinal tolerance in a nutritional intervention trial. This preliminary study manifests the need to work with a multidisciplinary approach in the development of functional foods. In this context, the present study provides information regarding the risk/benefit balance of the combined use of coffee cascara and inulin to obtain a food containing ingredients (glucosidase inhibitors and dietary fiber) with the potential to modulate appetite, improve gastrointestinal health, and provide a new flavor with good sensory acceptance. In conclusion, a novel yogurt formulation containing coffee-cascara extract (10 mg/mL) and 3% inulin that is well tolerated and sensory accepted with the nutritional claim “source of dietary fiber” has been obtained. The yogurt can be included in the daily diet for contributing to the intake of this nutrient. 

## Figures and Tables

**Table 1:** Measurements of pH and titratable acidity of yogurt with cascara extract (Y0), yogurt with cascara extract and 3% inulin (Y3), yogurt with cascara extract and 7% inulin (Y7), and yogurt with cascara extract and 13% inulin (Y13).

	Y0	Y3	Y7	Y13
pH	4.59 ± 0.16 ^a^	4.57 ± 0.14 ^a^	4.57 ± 0.14 ^a^	4.48 ± 0.05 ^a^
Titratable acidity (g lactic acid/100g yogurt)	0.69 ± 0.04 ^ab^	0.74 ± 0.02 ^b^	0.71 ± 0.03 ^ab^	0.64 ± 0.01^a^

Values in each row with different letters differ significantly (Tukey test, ^a,b^ *p* < 0.05).

**Table 2:** Overall liking and acceptability of individual attributes evaluated by the participants (*n* = 45) for each yogurt treatment.

	Y0	Y3	Y7	Y13
Overall liking	5.47 ± 1.25 ^a^	5.82 ± 1.23 ^ab^	5.78 ± 1.39 ^ab^	6.44 ± 1.23 ^b^
Smell	6.42 ± 1.05 ^a^	6.4 ± 1.03 ^a^	6.6 ± 1.45 ^a^	6.467 ± 1.11 ^a^
Appearance	6.07 ± 1.21 ^a^	5.91 ± 1.35 ^a^	5.89 ± 1.51 ^a^	6.47 ± 1.39 ^a^
Texture	4.89 ± 1.60 ^a^	5.31 ± 1.44 ^a^	5.69 ± 1.65 ^a^	6.76 ± 1.41^b^
Taste	5.2 ± 1.47 ^a^	5.38 ± 1.55 ^a^	5.71 ± 1.61 ^ab^	6.04 ± 1.69 ^b^

Values are represented as mean ± SD. Values with different letters are significantly different (^a,b^ *p* < 0.05).

**Table 3:** Frequency of occurrence and total and individual scores of gastrointestinal symptoms in the 2 and 24 h following consumption of the yogurt treatments in healthy adults (*n* = 45).

	Yogurt Formulation			
	Y0	Y3	Y7	Y13
***Bloating***				
No symptoms	31	32	23	19
More than usual	13	11	13	16
Much more than usual	1	2	9	9
Extremely more than usual	0	0	0	1
Significance	*-*	*ns*	*	**
Individual score	0.33 ± 0.52	0.33 ± 0.56	0.69 ± 0.79	0.82 ± 0.83
***Nausea***				
No symptoms	42	43	42	45
More than usual	2	2	3	0
Much more than usual	1	0	0	0
Extremely more than usual	0	0	0	0
Significance	*-*	*ns*	*ns*	*ns*
Individual score	0.09 ± 0.36	0.04 ± 0.21	0.07 ± 0.25	0.00 ± 0.00
***Flatulence***				
No symptoms	38	34	25	24
More than usual	7	9	14	14
Much more than usual	0	2	6	5
Extremely more than usual	0	0	0	2
Significance	*-*	*ns*	**	**
Individual score	0.16 ± 0.37	0.29 ± 0.55	0.58 ± 0.72	0.67 ± 0.85
***Abdominal pain***				
No symptoms	41	38	38	32
More than usual	4	6	6	11
Much more than usual	0	1	1	2
Extremely more than usual	0	0	0	0
Significance	*-*	*ns*	*ns*	*
Individual score	0.09 ± 0.29	0.18 ± 0.44	0.18 ± 0.44	0.33 ± 0.56
***Diarrhea***				
No symptoms	41	42	39	38
More than usual	4	3	4	6
Much more than usual	0	0	2	1
Extremely more than usual	0	0	0	0
Significance	*-*	*ns*	*ns*	*ns*
Individual score	0.09 ± 0.37	0.07 ± 0.25	0.18 ± 0.49	0.18 ± 0.44
***Constipation***				
No symptoms	40	42	42	41
More than usual	5	2	2	3
Much more than usual	0	1	1	1
Extremely more than usual	0	0	0	0
Significance	-	*n*s	*n*s	*n*s
Individual score	0.11 ± 0.32	0.09 ± 0.36	0.09 ± 0.36	0.11 ± 0.38
***GI rumbling***				
No symptoms	39	37	34	28
More than usual	6	8	9	11
Much more than usual	0	0	2	6
Extremely more than usual	0	0	0	0
Significance	-	*n*s	*n*s	*
Individual score	0.13 ± 0.34	0.18 ± 0.39	0.29 ± 0.55	0.51 ± 0.73
***Total score***	1.00	1.18	2.07	2.62

The results are expressed as frequency of occurrence of individual gastrointestinal symptoms. Differences between the occurrence of individual gastrointestinal symptoms for the different yogurt samples were analyzed using a Chi-square test comparing the frequencies reported for Y3, Y7, and Y13 to those reported for Y0. * *p* < 0.05; ** *p* < 0.01; *n*s = not significant.

**Table 4:** Stool frequency and consistency within 2 to 24 h following the consumption of the yogurt treatments in healthy adults (*n* = 45).

	Yogurt formulation			
	Y0	Y3	Y7	Y13
***Stool frequency*** ^1^	1.47 ± 0.79	1.36 ± 0.68	1.76 ± 1.07	1.51 ± 0.79
Significance	-	*n*s	*n*s	*n*s
***Stool consistency*** ^2^				
Constipation	3	2	3	2
Normal	32	35	30	28
Diarrhea	7	4	10	14
Significance	-	*n*s	*n*s	*n*s

^1^ Stool frequency per participant is expressed as mean ± sd. Chi-square comparisons were made between relative frequencies of Y0 and the rest of the treatments (Y3, Y7, Y13). ^2^ Stool consistency is expressed as the frequency reported for each stool consistency type for the different yogurt treatments. Differences between the occurrence of stool consistency types for the different yogurt samples were analyzed using a Chi-square test comparing the frequencies reported for Y3, Y7 and Y13 to those reported for Y0.

## Supplementary Materials

The following are available online at https://www.mdpi.com/2072-6643/12/3/627/s1, Figure S1: Effect on α-glucosidase activity is represented by dose-response curves of the standard inhibitor acarbose (0.06 µg/mL–6.5 mg/mL) and coffee-cascara byproduct (0.01–4 mg/mL). Values represent mean ± standard deviation. This includes a duplicate of sample preparation and a triplicate of analysis.
