# Supplementary material for: Sensory Acceptance, Appetite Control and Gastrointestinal Tolerance of Yogurts Containing Coffee-Cascara Extract and Inulin
Source: Nutrients. 2020 Feb 27;12(3):627. doi: 10.3390/nu12030627 (PMC7146162; doi:10.3390/nu12030627)
Supplement: Supplementary file 1 [file nutrients-12-00627-s001.pdf]

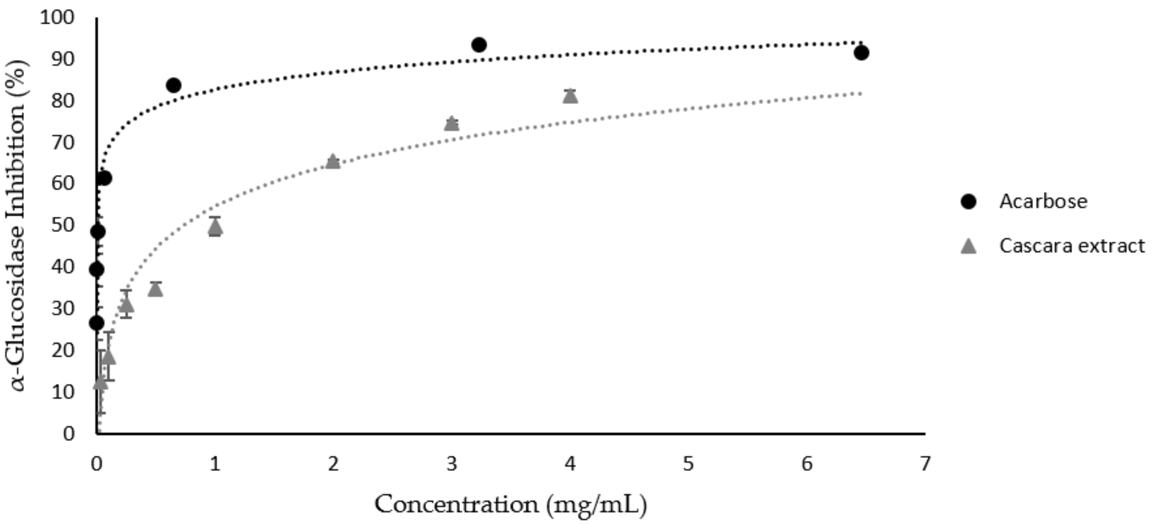

Figure S1. Effect on α-glucosidase activity is represented by dose-response curves of the standard inhibitor acarbose (0.06 µg/mL–6.5 mg/mL) and coffee-cascara byproduct (0.01–4 mg/mL). Values represent mean ± standard deviation. This includes a duplicate of sample preparation and a triplicate of analysis.
